# Effect of *Agrobacterium tumefaciens* on papaya whitefly *Trialeurodes variabilis* populations infesting papaya *Carica papaya*

**DOI:** 10.1038/s41598-026-55620-8

**Published:** 2026-05-30

**Authors:** Young-gyun Park, Matthew G. Hentz, Robert G. Shatters, Cindy L. McKenzie, Lance S. Osborne

**Affiliations:** 1 Department of Entomology and Nematology, Mid-Florida Research and Education Center, University of Florida, Apopka, FL USA; 2 Horticultural Research Laboratory, Subtropical Insects and Horticulture Research Unit, USDA-ARS, Fort Pierce, FL USA

**Keywords:** Bacteria, Crown gall disease, Hemiptera, Plant-microbe-herbivore interaction, Ecology, Ecology, Microbiology, Plant sciences

## Abstract

Crown gall disease, caused by *Agrobacterium tumefaciens*, is a tumor-inducing bacterial infection that alters host plant physiology. While this pathogen is extensively used in plant biotechnology, its role in shaping interactions with herbivorous insects remains poorly understood. In this study, we examined the effects of *A. tumefaciens* infection on the population dynamics of the papaya whitefly, *Trialeurodes variabilis*, and on the growth of papaya (*Carica papaya*) plants. Two greenhouse trials were conducted using healthy plants and plants in the *Agrobacterium* treatment. In the first trial, densities of whitefly adults were significantly higher in the *Agrobacterium* treatment. Similarly, in the second trial, densities of whitefly eggs and nymphs were significantly higher in the *Agrobacterium* treatment. Additionally, plants in the *Agrobacterium* treatment in the second trial exhibited reductions in plant height, canopy width, stem diameter, and SPAD index. Despite these changes, *A. tumefaciens*-infected plants remained suitable for supporting whitefly development. These results suggest that *A. tumefaciens* infection may promote whitefly population growth, highlighting how plant pathogens may influence plant-herbivore interactions. This study offers new insights into how plant–microbe interactions can influence herbivore populations and overall plant health.

**Supplementary Information:**

The online version contains supplementary material available at 10.1038/s41598-026-55620-8.

## Introduction

Papaya [*Carica papaya* L. (Brassicales: Caricaceae)] is a tropical plant native to Central America^1^. It is now widely cultivated in tropical regions and also grown in greenhouses in some temperate areas^1,2^. Major pests of papaya include the papaya fruit fly [*Toxotrypana curvicauda* Gerstaecker (Diptera: Tephritidae)], papaya whitefly [*Trialeurodes variabilis* (Quaintance) (Hemiptera: Aleyrodidae)], and two-spotted spider mite [*Tetranychus urticae* Koch (Acari: Tetranychidae)]^3–5^. These pests attack the fruits or leaves of the papaya, directly reducing yields or hindering plant growth, thereby causing economic losses. Interestingly, among these pests, the papaya whitefly may serve not only as a harmful agent but also as a potential component of biological control strategies^4^. Recently, papaya plants infested with papaya whitefly have gained attention as banker plants for maintaining natural enemies in greenhouses, and this approach has been actively studied and promoted in Florida, USA^4,6,7^. Therefore, papaya holds considerable value not only as a crop but also as a banker plant.

Crown gall disease is a plant disease caused by the gram-negative bacterium *Agrobacterium tumefaciens* (Smith and Townsend) (Hyphomicrobiales: Rhizobiaceae)^8,9^. The pathogen infects hundreds of plant species across numerous plant families, including economically important crops such as apple, cherry, grapevine, papaya, tomato, and walnut^8–10^. Infection results in tumor-like galls that form primarily on plant stems and disrupt normal plant growth and physiology, often leading to reduced vigor, stunted growth, and, in severe cases, plant death^8–10^. The bacterium typically establishes through wounds on plants; thus, preventive measures such as disinfecting farming tools are essential for managing crown gall disease^8,9^.

For herbivorous insects, the health condition of the host plant is a key factor influencing population size^11^. It is generally known that diseased host plants have a negative impact on the survival and reproduction of herbivorous insects^12,13^. However, Costa et al.^14^ reported that pumpkin (*Cucurbita maxima* Duchesne) infected with the watermelon curly mottle strain of squash leaf curl virus did not affect the fecundity of *B. tabaci*, but significantly increased egg-to-adult survival, from 52% on control plants to 75% on virus-infected plants. Furthermore, McKenzie et al.^15^ reported increases in *B. tabaci* egg (2.5-fold) and nymph (4.5-fold) numbers in tomato mottle virus-infected tomato plants compared to virus-uninfected tomato plants at 56 days after whitefly infestation. These cases suggests that plant pathogens do not always negatively affect insects; the outcomes may vary depending on specific interactions among the pathogen, host plant, and insect. Nonetheless, unlike viruses and fungi, little is known about how plant-pathogenic bacteria affect herbivorous insect populations, which motivated the present study.

In this study, we investigated whether crown gall disease influences the population size of papaya whitefly. To address this question, we compared the population dynamics of the whitefly on healthy and crown gall–infected papaya plants. We also measured plant parameters (e.g., canopy width, leaf number, plant height, and stem diameter) to evaluate how crown gall disease affects the fitness of papaya plants infested with *T. variabilis*. Results are discussed in the context of plant-microbe-herbivore interactions and their ecological implications.

## Materials and methods

### Papaya whitefly rearing

Adult males and females of *T. variabilis* were obtained from a laboratory colony maintained at the U.S. Horticultural Research Laboratory, Fort Pierce, FL, USA. The colony originated from a population collected in Apopka, FL, USA (GenBank accession no. KP032217) and has been continuously reared on papaya (*Carica papaya* cv. ‘Caribbean Red’) following the methods described by Xiao et al.^4^. All life stages were maintained on intact host plants by serial transfer in a walk-in environmental chamber under controlled conditions (27 ± 2 °C, 50 ± 10% RH, 16:8 h L: D photoperiod), without cages.

## Bacteria cultivation

One day prior to papaya inoculation with *A. tumefaciens*, a liquid bacterial culture was prepared. Ten milliliters of standard LB broth (prepared as directed; Alpha Teknova Inc., Hollister, CA, USA) was added to a sterile 50 mL Cellstar conical tube (Greiner Bio-One, Monroe, NC, USA). A 10 µL aliquot of a glycerol stock of *A. tumefaciens* (USDA, ARS, USHRL gall-inducing isolate #29 wild type, equivalent to isolate T28/73 wild type) was added, along with 10 µL of a 0.1% kanamycin solution^16^. Kanamycin was included in the culture medium to maintain selection for the bacterial strain. Several culture tubes were prepared and incubated at 28 °C with shaking at 180 rpm for 24 h in an incubator (C24 Incubator Shaker, Brunswick Scientific Classic Series, Edison, NJ, USA). The following day, the cultures were centrifuged at 5,000 rpm for 10 min at 25 °C. The supernatant was removed, and the bacterial pellet was resuspended in 25 mL of sterile 20% magnesium chloride MES buffer (100 mM MgCl₂ + 100 mM MES, mixed in equal parts and diluted in sterile H_2_O). The suspension was centrifuged again (5,000 rpm, 10 min), and the supernatant was discarded. The resulting pellet was resuspended in 10 mL of fresh 20% buffer. The optical density (OD) of the bacterial suspension was measured at 600 nm using a spectrophotometer (Jenway 7205 UV/Visible Spectrophotometer, Cole Palmer Ltd., Stone, Staffs, UK). Sterile buffer was added to adjust the OD₆₀₀ to a final value of 1.0. Immediately thereafter, the bacterial suspension was used to inoculate the papaya plants.

## Papaya plant cultivation

Papaya seeds were harvested from locally purchased fruits of *Carica papaya* ‘Caribbean Red’ obtained from a grocery store in Florida, USA. A total of 120 papaya seeds were individually sown into peat pellets (Jiffy Cubes; Jiffy Products of America Inc., Lorain, OH, USA) and placed in a cloth cage for germination on May 8, 2023. Each of the 120 seedlings at the two- to three-leaf stage was transplanted into a pot (approximately 3.79 L) on June 5. The seedlings were grown in a greenhouse until they reached the five- to six-leaf stage. On July 6, approximately one week before inoculation, the plants were treated with a fungicide (Azoxystrobin, Abound at 1.25 mL/L; Syngenta U.S., Greensboro, NC, USA) to prevent powdery mildew.

## Experiment

**Fig. 1:**
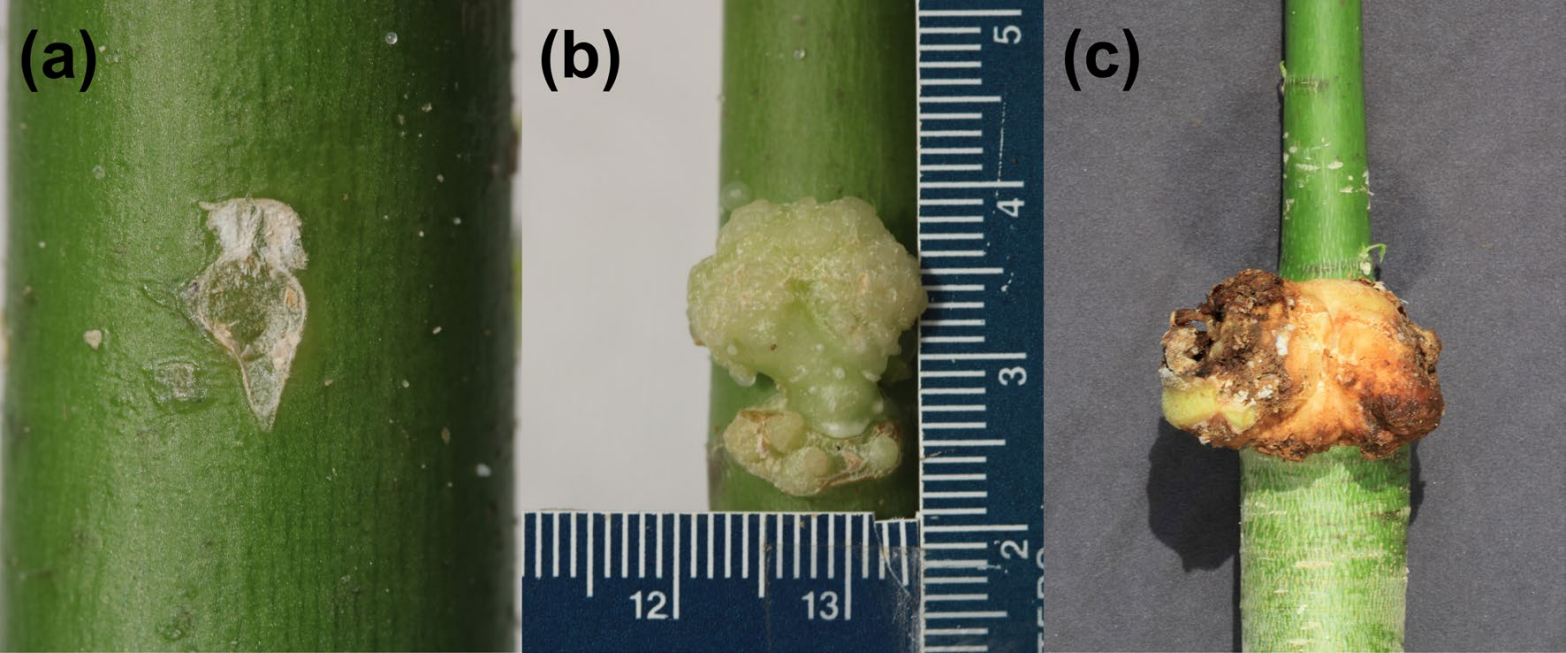
Papaya stem at the inoculation site from plants used in the experiment. (**a**) Control plant 20 days after buffer inoculation. (**b**) *Agrobacterium* treatment 20 days after inoculation. (**c**) *Agrobacterium* treatment 91 days after inoculation.

On July 12, a 1.5-mm-deep hole was made at a 45-degree angle on the stem of each of the 120 papaya plants, at a height of 15 cm above the soil line and at the 5- to 6-leaf stage, using a biopsy punch (1.5 mm Integra Miltex Disposable Biopsy Punch with Plunger, Integra LifeSciences Production Corp., Mansfield, CA, USA). Ten microliters (10 µL) of *A. tumefaciens* isolate #29 suspension was pipetted into the hole of each of 60 plants as the *Agrobacterium* treatment, and 10 µL of magnesium chloride MES buffer (Sigma-Aldrich, St. Louis, MO, USA) was pipetted into the hole of the other 60 plants as the control. The puncture site was immediately wrapped with Parafilm (Bemis Company, Neenah, WI, USA). Infection by *A. tumefaciens* was determined based on the development of crown galls on inoculated plants, whereas no gall formation was observed in the control plants (Fig. 1).

On August 8, gall formation was observed in about 20 of the 60 plants in the *Agrobacterium* treatment group. Twenty of these plants were selected, along with 20 similarly sized plants from the control group, and assigned to the first trial. The 20 plants from each treatment were divided into four groups of five and transferred to separate 32 × 32 mesh Lumite^®^ screen field cages with a zipper entrance (1.8 × 1.8 × 1.8 m) (BioQuip, Rancho Dominguez, CA, USA). Thus, each treatment included four replicates, with each replicate consisting of five plants enclosed in a mesh cage. On August 14 and 21, *T. variabilis* adults were released into all cages in the first trial to infest papaya plants, at a rate of five adults per leaf on each date, for a total of two releases.

On August 15, gall formation was confirmed in more than 20 additional plants from the *Agrobacterium* treatment group that had not shown gall formation on August 8. Twenty of these plants were selected and, along with 20 similarly sized plants from the control group, were assigned to the second trial. As in the first trial, each treatment in the second trial was divided into four replicates (five plants per replicate) and transferred to separate cages. On August 25 and September 1, *T. variabilis* adults were released into all cages in the second trial to infest papaya plants, at a rate of five adults per leaf on each date, for a total of two releases. Thus, the primary difference between the first and second trials was the timing of whitefly infestation: five and six weeks after inoculation in the first trial, and six and seven weeks after inoculation in the second trial because of delayed gall formation. In total, gall formation was confirmed in more than 40 plants among the 60 inoculated plants. Plants that did not develop visible galls were not included in the trials because the objective was to examine the effects of crown gall disease on whitefly populations. In addition, there were slight differences in the timing of observations between the two trials (Table S1).

Whitefly counts were conducted on all five plants in each replicate. For immature whitefly counts, including eggs and nymphs, a leaf disc was sampled from one leaf at each canopy level (upper, middle, and lower) of each plant using No. 8 brass cork borers (1 cm²; Boekel Scientific, Feasterville-Trevose, PA, USA). Thus, 15 leaf discs per replicate were used to count whitefly eggs and nymphs under a microscope in the laboratory. To assess adult *T. variabilis* density, one leaf from each of the upper, middle, and lower canopy levels was randomly selected from each plant, and the number of adult whiteflies on each leaf was counted with the naked eye. Accordingly, 15 leaves per cage (i.e., per replicate) were examined for adult whiteflies. Whitefly counts began one week after the final infestation of the plants (August 28 in the first trial; September 8 in the second trial) and continued at approximately weekly intervals for a total of seven weeks.

All five plants in each replicate were used to assess plant parameters, including number of leaves, plant height, canopy width, stem diameter, and SPAD (Soil Plant Analysis Development) index. In the *Agrobacterium* treatment, additional measurements were taken for gall profile, including width and height. The number of leaves was determined by counting fully expanded leaves. Plant height was measured from the soil surface to the top of the plant using a meter stick. Canopy width was measured by viewing the plant from above and recording dimensions in two directions: North to South (N–S) and East to West (E–W), also using a meter stick. Stem diameter was measured at three positions: the treated spot (inoculation point), 5 cm above, and 5 cm below the inoculation point. SPAD indices were obtained from five leaves located in the middle canopy of each selected plant using a SPAD-502 Plus chlorophyll meter (Konica Minolta Sensing, Inc., Tokyo, Japan). Gall width was measured in both N–S and E–W directions, and gall height was measured from the main stem using a ruler. During the first trial, the number of leaves was measured 11 times; plant height and canopy width were measured 5 times; stem diameter at the inoculation point was measured 4 times; stem diameters (5 cm above and below the inoculation point) and SPAD index were measured 13 times; and gall measurements were taken 9 times starting from gall formation (Table S1). In the second trial, the number of leaves was measured 11 times; plant height and canopy width were measured 5 times; stem diameter at the inoculation point was measured 4 times; stem diameters (5 cm above and below the inoculation point) and SPAD index were measured 15 times; and gall measurements were taken 10 times starting from gall formation (Table S1).

### Data analysis

For each observation date, the mean values of whitefly numbers and plant parameters per replicate (cage) were calculated and used in the data analysis. Repeated measurements of mean values for whitefly counts and plant parameters, except for gall measurements, in each trial were compared between treatments using a generalized linear mixed model (GLMM) with a Gaussian distribution and identity link function in PROC GLIMMIX in SAS^17^. Treatment and observation date were included as fixed effects, and replicate was treated as the experimental unit for repeated measurements. Repeated measurements of each gall measurement from 0 to 8 weeks after gall formation were compared between the two trials using a GLMM with a Gaussian distribution and identity link function in PROC GLIMMIX in SAS^17^. Trial was included as a fixed effect, and observation date was included as a random effect because the timing of observations differed between the two trials. Model assumptions were evaluated by inspecting residual plots.

## Results

### Papaya whitefly: egg

**Fig. 2:**
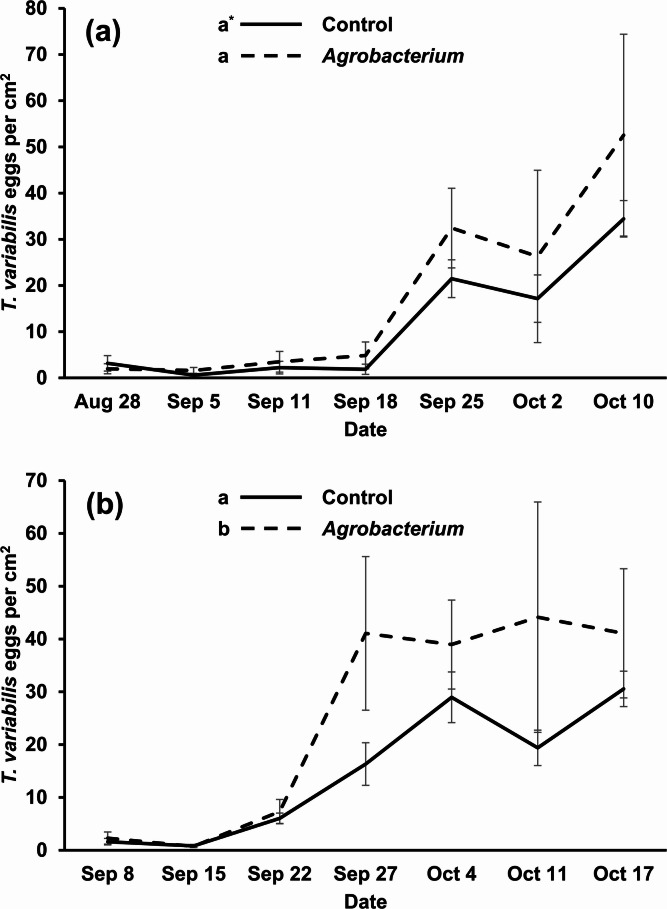
Mean (± SE) weekly densities (number per cm^2^) of *Trialeurodes variabilis* eggs in the first trial (**a**) and second trial (**b**). ^*^Curves bearing the same letter in each graph legend are not significantly different (PROC GLIMMIX, least squares means comparison, α = 0.05; ‘a’ is higher than ‘b’).

In the first trial, the density of *T. variabilis* eggs varied significantly over time, with no treatment × observation date interaction (Table 1). The mean peak densities of *T. variabilis* eggs in the control and *Agrobacterium* treatments were 34.4 and 52.5 eggs per cm^2^ on October 10, respectively (Fig. 2a). However, there was no significant difference in the overall densities of *T. variabilis* eggs between the control and *Agrobacterium* treatments (Table 1). Estimated treatment differences and their 95% confidence intervals from the GLMM least-squares means comparisons are provided in Table S2.

**Table 1:** GLMM results by category for whitefly and papaya plant measurements.

Category	Source	First trial			Second trial		
		*F*	*df*	*P*	*F*	*df*	*P*
Whitefly: Eggs	Treatment	1.83	1, 42	0.184	5.28	1, 42	0.027
	Date	7.77	6, 42	< 0.001	7.31	6, 42	< 0.001
	Interaction	0.34	6, 42	0.909	0.83	6, 42	0.551
Whitefly: Nymphs	Treatment	3.78	1, 42	0.059	7.36	1, 42	0.010
	Date	10.66	6, 42	< 0.001	17.79	6, 42	< 0.001
	Interaction	1.43	6, 42	0.225	1.46	6, 42	0.215
Whitefly: Adults	Treatment	6.28	1, 42	0.016	2.43	1, 42	0.126
	Date	37.31	6, 42	< 0.001	21.96	6, 42	< 0.001
	Interaction	1.66	6, 42	0.154	0.22	6, 42	0.968
Papaya plant: Number of leaves	Treatment	1.63	1, 66	0.206	7.19	1, 66	0.009
	Date	21.31	10, 66	< 0.001	18.04	10, 66	< 0.001
	Interaction	0.22	10, 66	0.993	0.82	10, 66	0.613
Papaya plant: Plant height	Treatment	0.55	1, 30	0.462	19.34	1, 30	< 0.001
	Date	156.28	4, 30	< 0.001	84.24	4, 30	< 0.001
	Interaction	0.10	4, 30	0.981	1.13	4, 30	0.363
Papaya plant: Canopy width (N-S)	Treatment	0.36	1, 30	0.556	9.24	1, 30	0.005
	Date	90.82	4, 30	< 0.001	140.11	4, 30	< 0.001
	Interaction	0.38	4, 30	0.818	1.53	4, 30	0.218
Papaya plant: Canopy width (E-W)	Treatment	0.05	1, 30	0.831	13.95	1, 30	< 0.001
	Date	87.40	4, 30	< 0.001	191.04	4, 30	< 0.001
	Interaction	0.13	4, 30	0.970	0.97	4, 30	0.438
Papaya plant: Stem diameter (inoculation point)	Treatment	0.14	1, 24	0.708	9.48	1, 24	0.005
	Date	510.93	3, 24	< 0.001	348.17	3, 24	< 0.001
	Interaction	0.63	3, 24	0.600	0.60	3, 24	0.619
Papaya plant: Stem diameter (5 cm above)	Treatment	48.24	1, 78	< 0.001	30.15	1, 90	< 0.001
	Date	272.49	12, 78	< 0.001	77.64	14, 90	< 0.001
	Interaction	0.65	12, 78	0.793	0.37	14, 90	0.980
Papaya plant: Stem diameter (5 cm below)	Treatment	1.58	1, 78	0.213	3.65	1, 90	0.059
	Date	444.34	12, 78	< 0.001	250.29	14, 90	< 0.001
	Interaction	2.85	12, 78	0.003	1.02	14, 90	0.438
Papaya plant: SPAD	Treatment	0.59	1, 78	0.447	8.43	1, 90	0.005
	Date	25.44	12, 78	< 0.001	14.24	14, 90	< 0.001
	Interaction	0.34	12, 78	0.980	1.02	14, 90	0.441

In the second trial, the density of *T. variabilis* eggs significantly differed by treatment and observation date; however, there was no significant interaction between treatment and observation date (Table 1). The overall densities of *T. variabilis* eggs were significantly higher in the *Agrobacterium* treatment than in the control (Table 1; Fig. 2b). The mean peak density of *T. variabilis* eggs reached 44.1 eggs per cm² on October 11 in the *Agrobacterium* treatment, compared to 30.6 eggs per cm² on October 17 in the control.

## Papaya whitefly: nymph

**Fig. 3:**
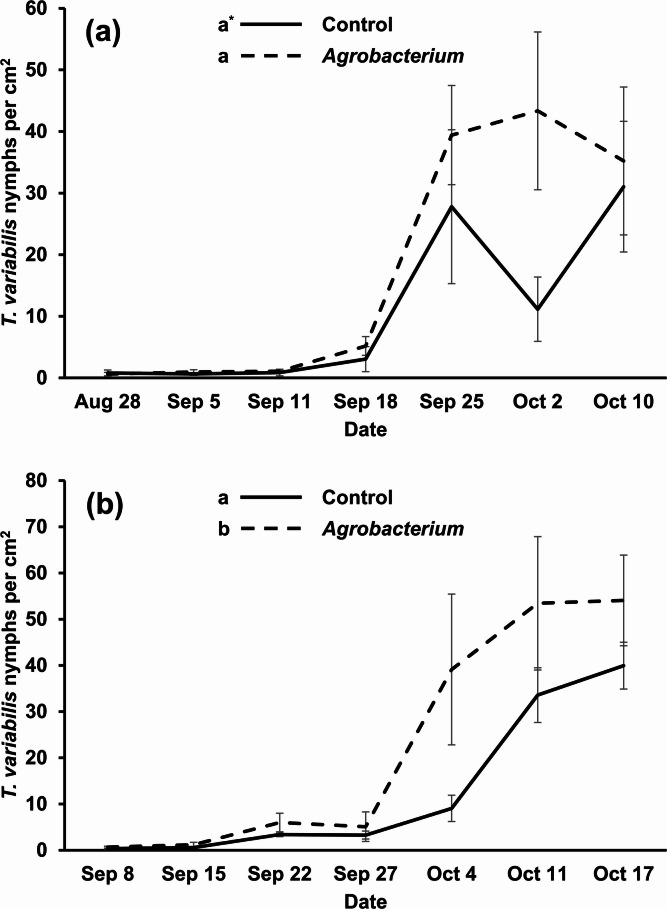
Mean (± SE) weekly densities (number per cm^2^) of *Trialeurodes variabilis* nymphs in the first trial (**a**) and second trial (**b**). ^*^Curves bearing the same letter in each graph legend are not significantly different (PROC GLIMMIX, least squares means comparison, α = 0.05; ‘a’ is higher than ‘b’).

In the first trial, the density of *T. variabilis* nymphs varied significantly over time, with no treatment × observation date interaction (Table 1). The mean peak densities of *T. variabilis* nymphs were 31.0 nymphs per cm² in the control on October 10 and 43.4 nymphs per cm² in the *Agrobacterium* treatment on October 2 (Fig. 3a). However, there was no significant difference in the overall densities of *T. variabilis* nymphs between the control and *Agrobacterium* treatments (Table 1).

In the second trial, the density of *T. variabilis* nymphs significantly differed by treatment and observation date; however, there was no significant interaction between treatment and observation date (Table 1). The overall densities of *T. variabilis* nymphs were significantly higher in the *Agrobacterium* treatment than in the control (Table 1; Fig. 3b). The mean peak density of *T. variabilis* nymphs was observed on October 17, with 54.1 nymphs per cm² in the *Agrobacterium* treatment and 40.0 nymphs per cm² in the control.

### Papaya whitefly: adult

**Fig. 4:**
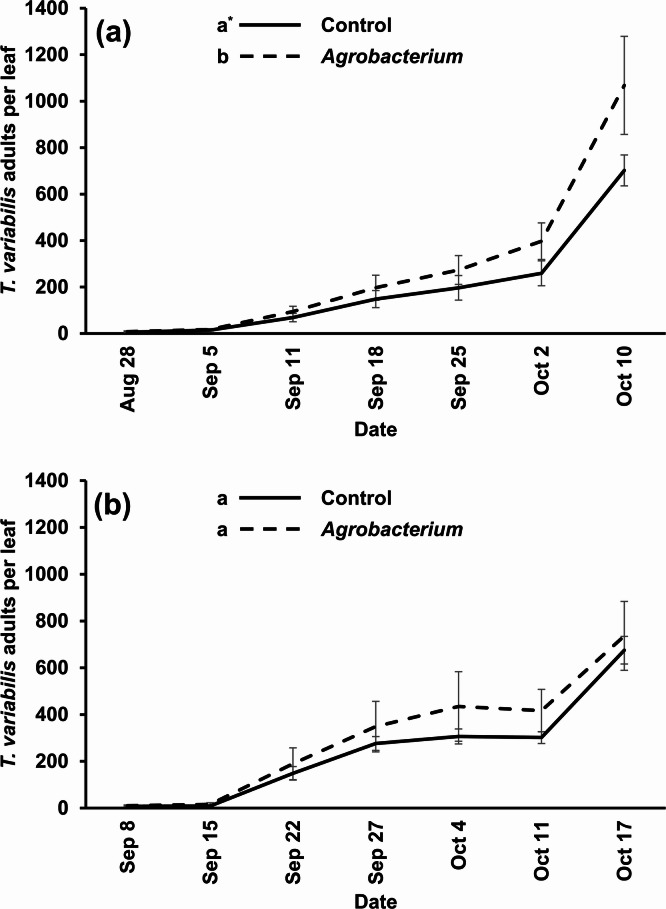
Mean (± SE) weekly densities (number per leaf) of *Trialeurodes variabilis* adults in the first trial (**a**) and second trial (**b**). ^*^Curves bearing the same letter in each graph legend are not significantly different (PROC GLIMMIX, least squares means comparison, α = 0.05; ‘a’ is higher than ‘b’).

In the first trial, the density of *T. variabilis* adults was significantly affected by treatment and observation date, with no treatment × observation date interaction (Table 1). The overall densities of *T. variabilis* adults were significantly higher in the *Agrobacterium* treatment than in the control (Table 1; Fig. 4a). The mean peak densities of *T. variabilis* adults in the control and *Agrobacterium* treatments were 702.3 and 1068.2 whiteflies per leaf on October 10, respectively.

In the second trial, the density of *T. variabilis* adults significantly differed by observation date; however, there was no significant interaction between treatment and observation date (Table 1). Differences of more than 100 whiteflies in mean values between treatments were observed on October 4 and 11; however, there was no significant difference in the overall densities of *T. variabilis* adults between the control and *Agrobacterium* treatments (Table 1; Fig. 4b).

### Papaya plant: number of leaves

**Fig. 5:**
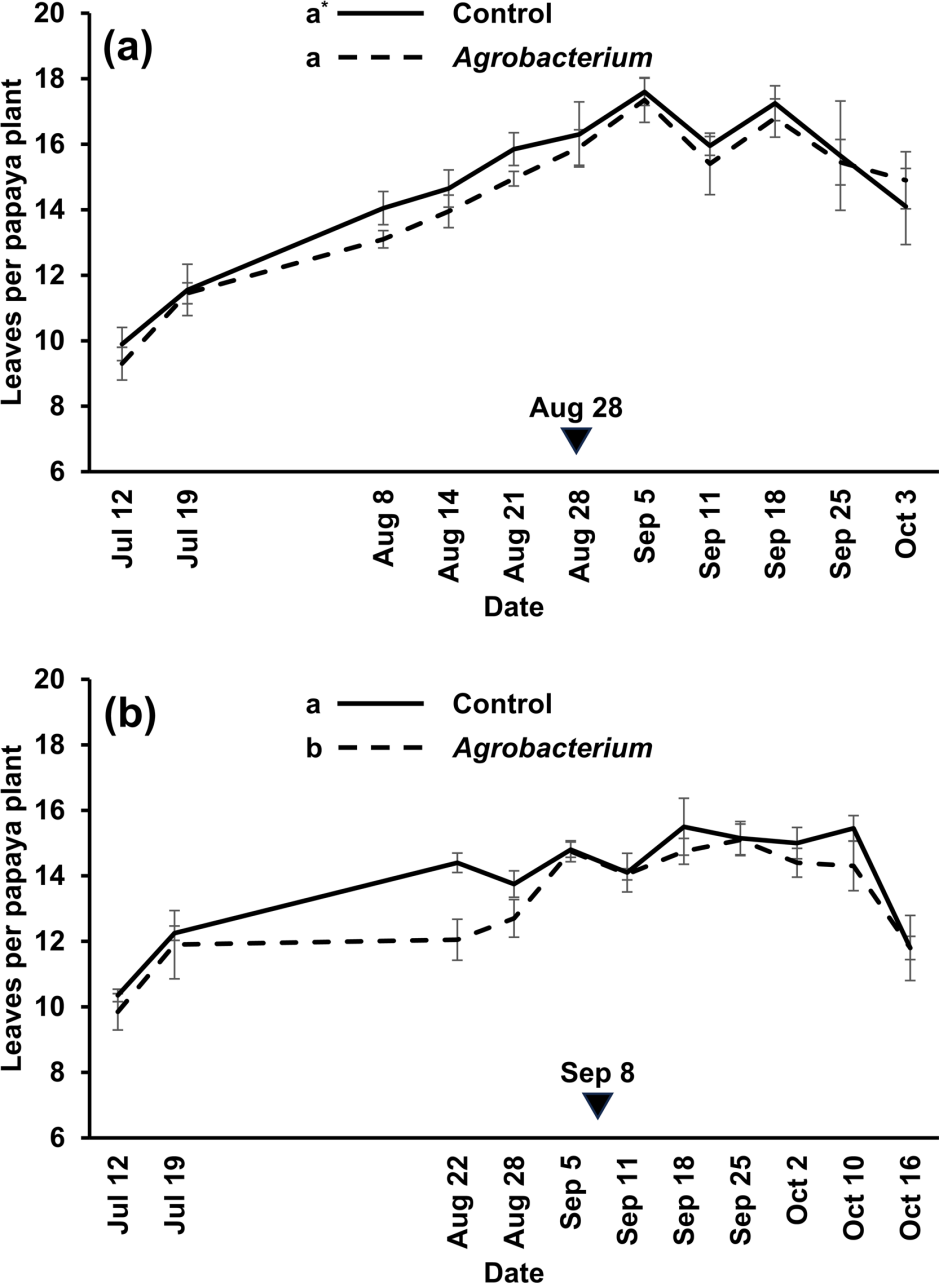
Mean (± SE) weekly leaf numbers of papaya plants in the first trial (**a**) and second trial (**b**). ▼: Whitefly observation start date. ^*^Curves bearing the same letter in each graph legend are not significantly different (PROC GLIMMIX, least squares means comparison, α = 0.05; ‘a’ is higher than ‘b’).

In both trials, the number of leaves in papaya plants varied significantly over time, with no treatment × observation date interaction (Table 1). The number of leaves in papaya plants was not significantly affected by treatment in the first trial; however, a significant effect was observed in the second trial (Table 1; Fig. 5). The overall number of leaves in the second trial was significantly higher in the control than in the *Agrobacterium* treatment (Fig. 5). On August 22, the difference in mean number of leaves between treatments was 2.4 leaves.

### Papaya plant: plant height

**Fig. 6:**
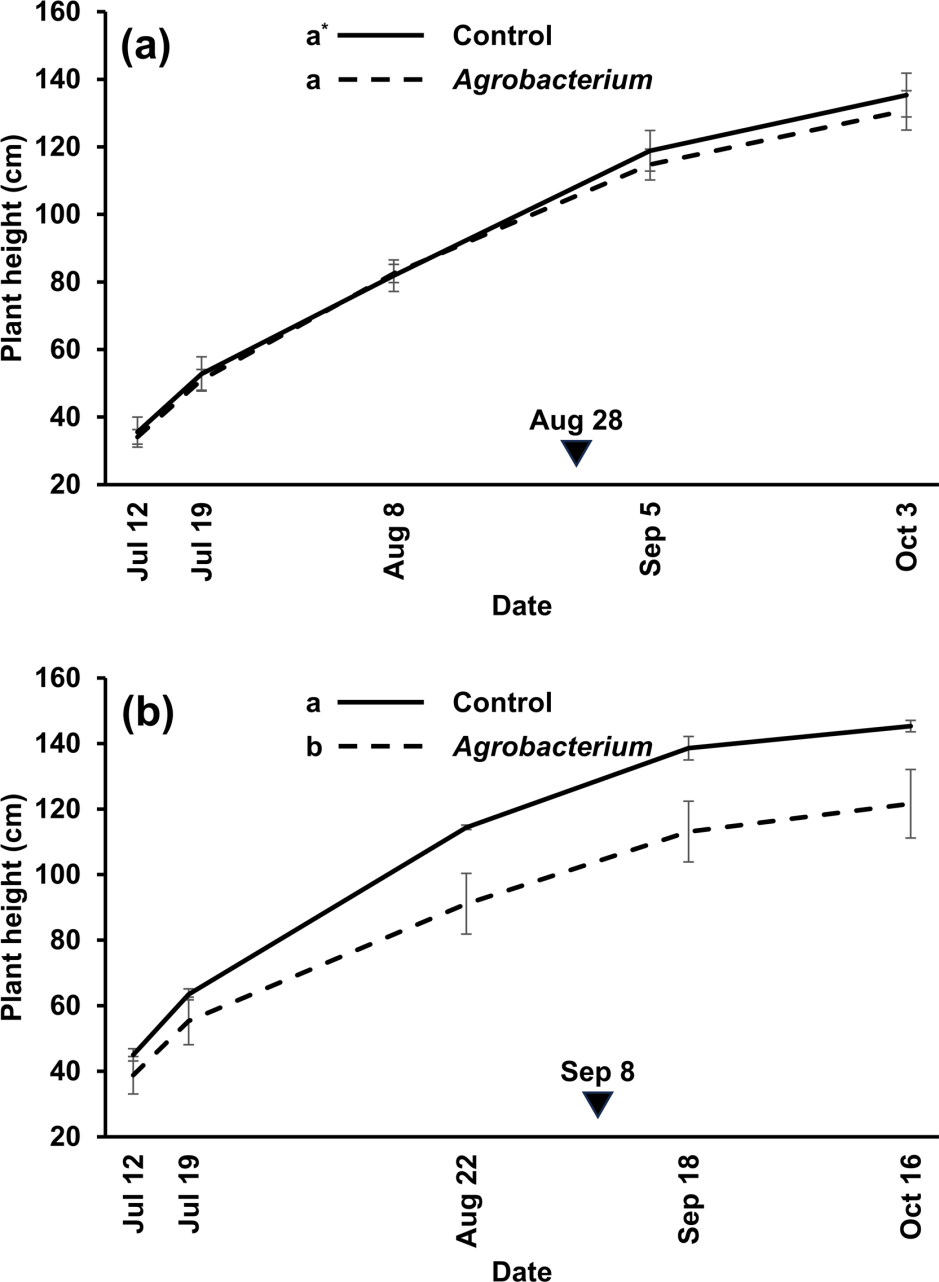
Mean (± SE) weekly heights (cm) of papaya plants in the first trial (**a**) and second trial (**b**). ▼: Whitefly observation start date. ^*^Curves bearing the same letter in each graph legend are not significantly different (PROC GLIMMIX, least squares means comparison, α = 0.05; ‘a’ is higher than ‘b’).

In both the first and second trials, the height of papaya plants was significantly affected by observation date, with no significant interaction between treatment and observation date (Table 1). In the first trial, there was no significant difference in plant height between the control and *Agrobacterium* treatments (Table 1; Fig. 6a). At the last observation date (October 3), plant heights in the control and *Agrobacterium* treatments were 135.3 cm and 130.8 cm, respectively.

However, in the second trial, plant height in the control was significantly higher than that in the *Agrobacterium* treatment (Table 1; Fig. 6b). On October 16, plant heights in the control and *Agrobacterium* treatments were 145.3 cm and 121.6 cm, respectively.

### Papaya plant: canopy width

**Fig. 7:**
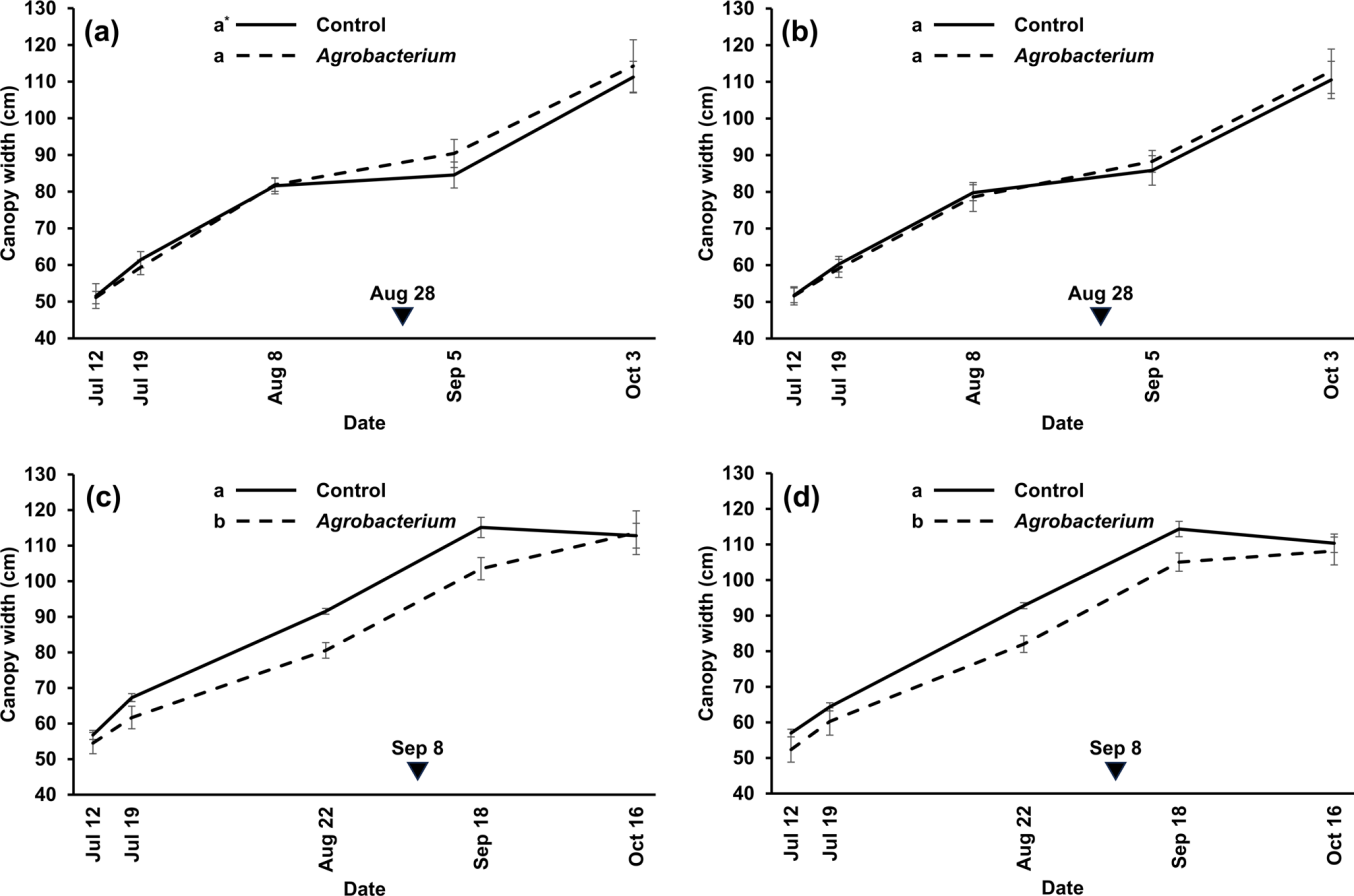
Mean (± SE) weekly canopy widths (cm) of papaya plants in the N–S (**a** first trial; c: second trial) and E–W direction (**b**: first trial; d: second trial). ▼: Whitefly observation start date. ^*^Curves bearing the same letter in each graph legend are not significantly different (PROC GLIMMIX, least squares means comparison, α = 0.05; ‘a’ is higher than ‘b’).

In both the first and second trials, the canopy width of papaya plants in both the north–south (N–S) and east–west (E–W) directions was significantly affected by observation date, with no significant interaction between treatment and observation date (Table 1). In the first trial, there was no significant difference in canopy width between the control and *Agrobacterium* treatments in either direction (Table 1; Fig. 7a, b).

However, in the second trial, canopy widths in both directions were significantly greater in the control than in the *Agrobacterium* treatment (Table 1; Fig. 7c, d). The greatest differences were observed on August 22 and September 18, with canopy widths differing by 11.0 to 11.6 cm (N–S) and 9.3 to 10.8 cm (E–W), respectively. However, by the final observation date on October 16, the differences had largely disappeared.

### Papaya plant: stem diameter

**Fig. 8:**
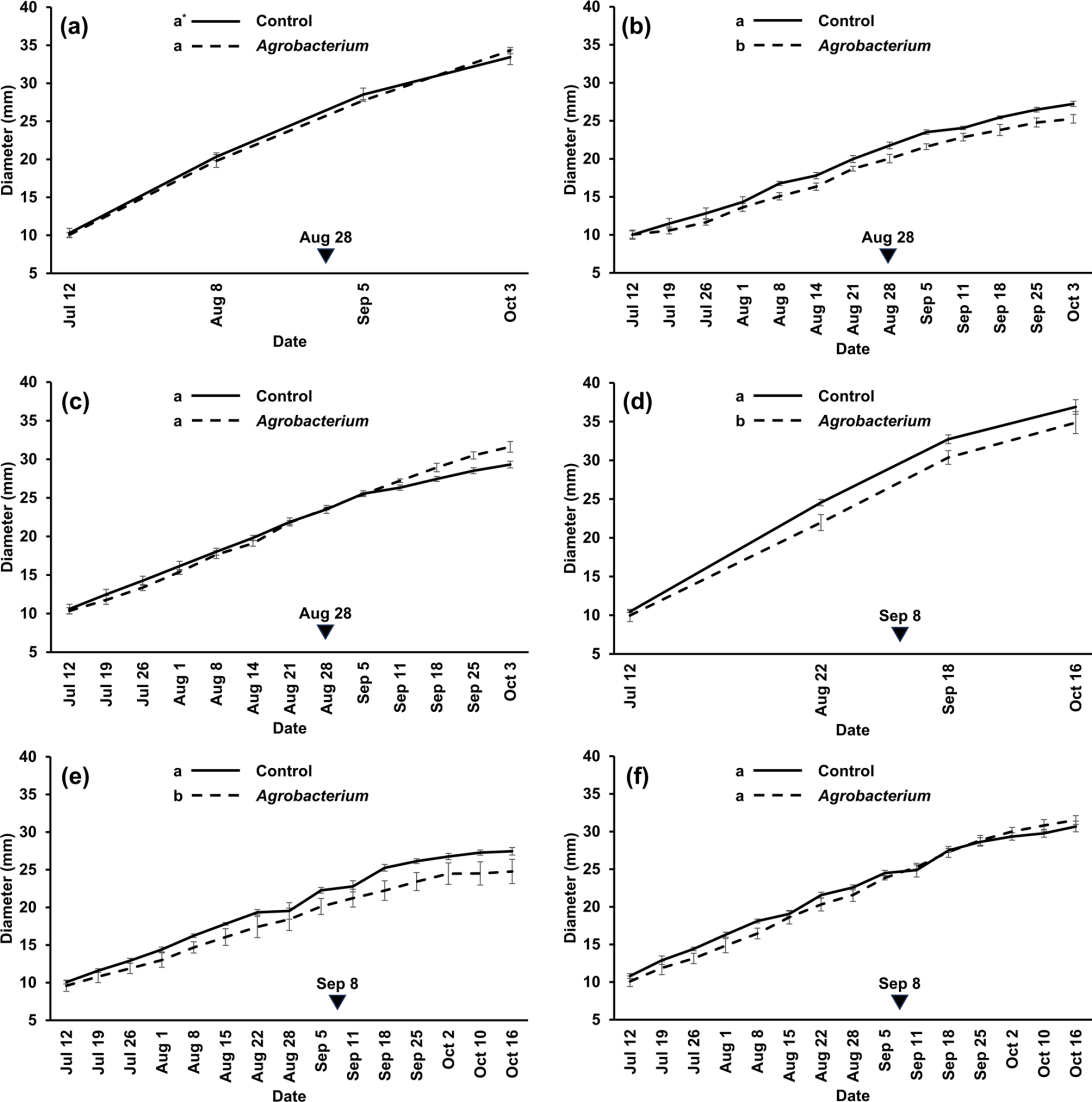
Mean (± SE) weekly stem diameters (mm) of papaya plants at the inoculation point (**a**: first trial; d: second trial), 5 cm above (**b**: first trial; e: second trial), and 5 cm below (c: first trial; f: second trial) the inoculation point. ▼: Whitefly observation start date. ^*^Curves bearing the same letter in each graph legend are not significantly different (PROC GLIMMIX, least squares means comparison, α = 0.05; ‘a’ is higher than ‘b’).

In the first trial, the stem diameter at the inoculation point was significantly affected by observation date but not by treatment, with no significant interaction between treatment and observation date (Table 1; Fig. 8a). The stem diameter 5 cm above the inoculation point was significantly affected by both treatment and observation date, with no significant interaction between the two (Table 1; Fig. 8b). This diameter was significantly greater in the control than in the *Agrobacterium* treatment, with a mean difference of 2.0 mm on October 3. The stem diameter 5 cm below the inoculation point was significantly affected by observation date but not by treatment, and there was a significant interaction between treatment and observation date (Table 1; Fig. 8c).

In the second trial, all three stem diameter measurements were significantly affected by observation date, with no significant interaction between treatment and observation date (Table 1). The stem diameters at the inoculation point and 5 cm above were both significantly greater in the control than in the *Agrobacterium* treatment (Table 1; Fig. 8d, e). At both points, the differences in mean diameter between treatments on September 18 ranged from 2.4 to 3.0 mm. However, there was no significant difference in stem diameter 5 cm below the inoculation point (Table 1; Fig. 8f).

### Papaya plant: SPAD

**Fig. 9:**
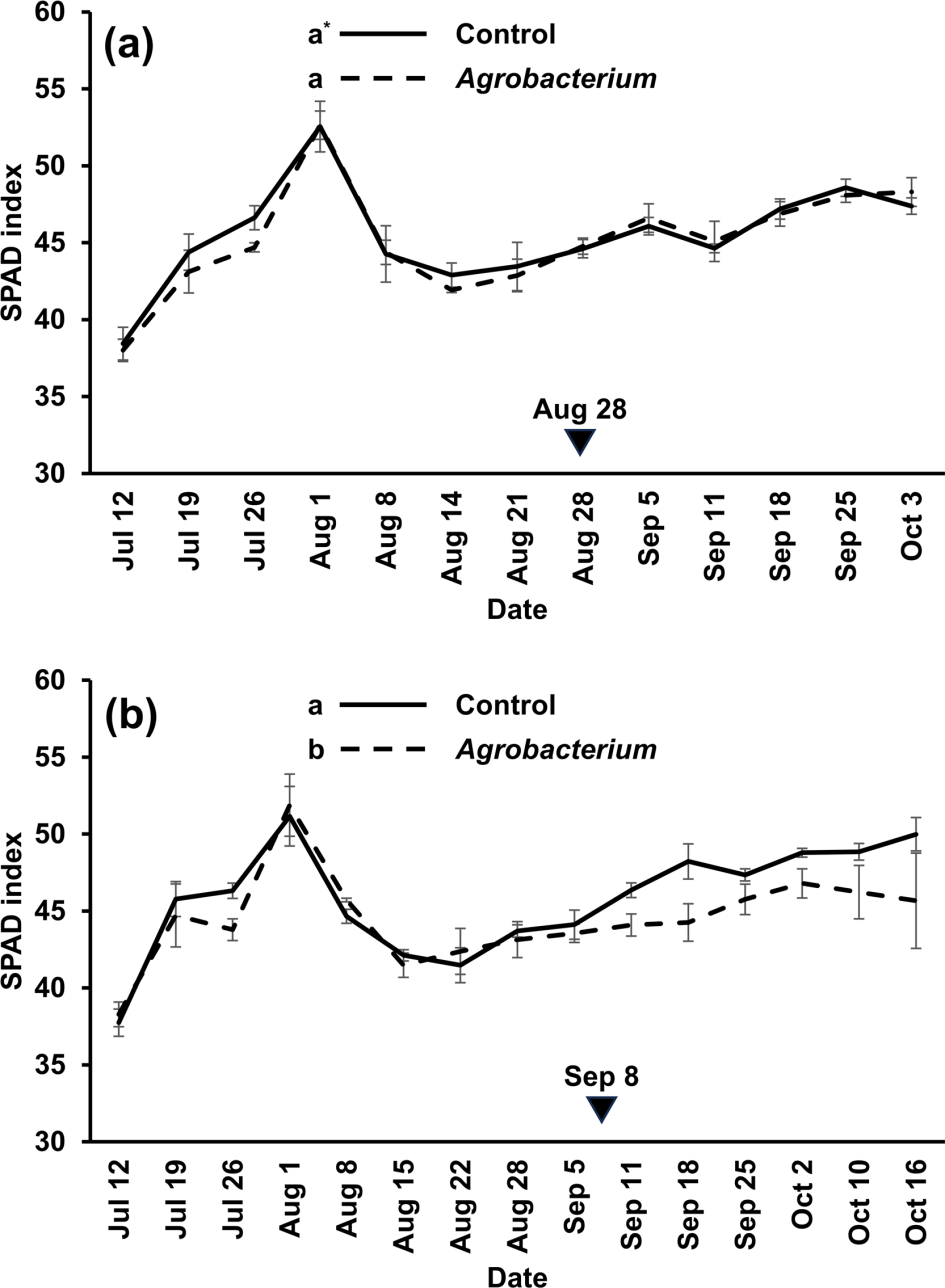
Mean (± SE) weekly SPAD indices of papaya leaves in the first trial (**a**) and second trial (**b**). ▼: Whitefly observation start date. ^*^Curves bearing the same letter in each graph legend are not significantly different (PROC GLIMMIX, least squares means comparison, α = 0.05; ‘a’ is higher than ‘b’).

In the first trial, the SPAD index was significantly affected by observation date but not by treatment, with no significant interaction between observation date and treatment (Table 1; Fig. 9a). However, in the second trial, the SPAD index was significantly affected by both treatment and observation date, with no significant interaction between the two (Table 1; Fig. 9b). On October 16, the SPAD indices in the control and *Agrobacterium* treatments in the second trial were 50.0 and 45.7, respectively.

### Papaya plant: gall measurement

**Fig. 10:**
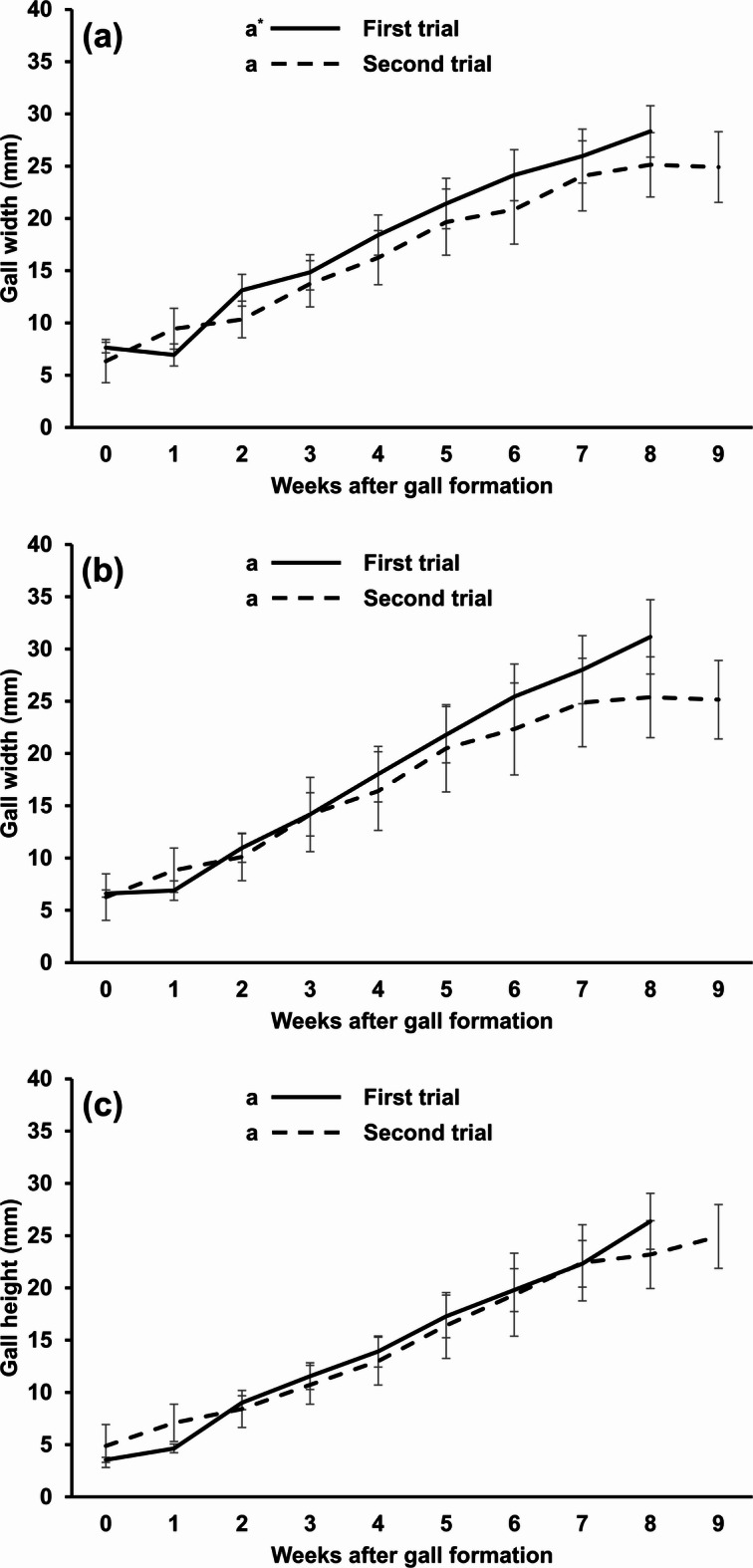
Mean (± SE) weekly gall measurements (mm) for the first and second trials: gall widths in the N–S direction (**a**) and E–W direction (**b**) and gall height (**c**). ^*^Curves bearing the same letter in each graph legend are not significantly different (PROC GLIMMIX, least squares means comparison, α = 0.05; ‘a’ is higher than ‘b’).

In both the first and second trials, galls continued to grow until the end of the experiment (Fig. 10), and there were no significant differences in any gall measurements after gall formation between the two trials (N–S direction: *F* = 0.77, *df* = 1, 70, *P* = 0.383; E–W direction: *F* = 0.50, *df* = 1, 70, *P* = 0.482; height: *F* = 0.03, *df* = 1, 70, *P* = 0.862). At 8 weeks after gall formation, gall widths in the N–S and E–W directions and gall height were 28.3, 31.2, and 26.4 mm in the first trial, and 25.1, 25.4, and 23.2 mm in the second trial, respectively.

## Discussion

In agriculture, *A. tumefaciens* is recognized as a plant pathogen in its natural state but has also been genetically modified as a tool for gene delivery in plants^18^. Research involving *A. tumefaciens* and insects has largely focused on the introduction of insect-resistance genes into plants^19–23^. To our knowledge, this is among the first studies to examine the effects of *A. tumefaciens* on the population growth of a herbivorous insect, offering insight into plant-pathogen-herbivore interactions involving *A. tumefaciens*. The present study demonstrated that inoculation with *A. tumefaciens* significantly affected the population density of the papaya whitefly, *T. variabilis*, as well as the growth parameters of infested papaya plants.

A significant increase in the densities of both immature stages, eggs and nymphs, was found in the *Agrobacterium* treatment in the second trial (Figs. 2b and 3b), with nymph densities also marginally higher in the *Agrobacterium* treatment in the first trial (Table 1; *P* = 0.059). In contrast to densities of eggs and nymphs, density of *T. variabilis* adult was significantly higher in the *Agrobacterium* treatment in the first trial, but not in the second trial (Fig. 4). Despite differences exceeding 32 whitefly nymphs per cm² on October 2 in the first trial (Fig. 3a) and 100 whitefly adults per leaf between treatments on October 4 and 11 in the second trial (Fig. 4b), statistical significance was not achieved, likely due to high within-treatment variation or a limited observation period^24^. Overall, despite variation in significance across trials and developmental stages, the mean densities of eggs, nymphs, and adults were generally higher in the *Agrobacterium* treatment than in the control.

Thus, when considering all three developmental stages (eggs, nymphs, and adults) across both trials, the results would support the conclusion that *T. variabilis* populations were higher on plants inoculated with *A. tumefaciens*. Given that the experiments were conducted in enclosed cages, immigration of *T. variabilis* from outside the experimental area is unlikely to have influenced the observed densities. These findings suggest that *A. tumefaciens* infection may contributes to increased population growth rates of *T. variabilis*. Enhancement of the herbivore population by diseased plants, apart from cases of viral infection, is rarely reported, as plant infections are generally expected to reduce herbivore performance or alter host preference due to diminished plant condition^12,13,25^. However, *A. tumefaciens* induces gall formation by altering plant gene expression and physiology^26–28^, which may modify plant composition and resource allocation within infected plants. Because gall tissues can function as strong metabolic sinks, these changes may influence nutrient distribution and potentially create conditions favorable for phloem-feeding insects such as *T. variabilis*. In addition, *A. tumefaciens* can reduce the plant’s ability to produce certain defense-related compounds, making it more susceptible to pests^29^. However, the physiological mechanisms underlying this pattern were not directly examined in the present study. Further studies should investigate changes in the internal composition of *A. tumefaciens*-infected papaya plants and assess their effects on the life history traits of *T. variabilis*.

The *Agrobacterium* treatment also significantly affected papaya plants infested with *T. variabilis*. In the first trial, among all observed parameters, only stem diameter at 5 cm above the inoculation point differed significantly between treatments (Figs. 5, 6, 7, 8 and 9). The reduction in stem diameter above the inoculation point is presumably attributable to growth inhibition caused by the developing gall^8,9^. Despite gall formation, reduced stem diameter above the gall, and elevated whitefly densities in the treatment, plant growth remained largely comparable to that in the control.

In contrast, the second trial revealed marked differences between treatments in number of leaves, plant height, canopy width, stem diameter, and SPAD index (Figs. 5, 6, 7, 8 and 9). On August 22, the mean difference between treatments was approximately 1.1 leaves, but this difference gradually disappeared over time (Fig. 5b). Plant height was consistently greater in the control, with the maximum difference reaching 25.4 cm on September 18 (Fig. 6b). This trend remained consistent throughout the observation period. Canopy width also differed between treatments, particularly on August 22 and September 18, with the control exhibiting canopy widths approximately 10 cm greater. However, this difference decreased over time and was minimal by the final observation on October 16 (Fig. 7b). In our study, papaya plants were infested by *T. variabilis* between the fifth and seventh weeks after treatment inoculation, following the start of plant parameter measurements. Moreover, the symptoms observed, including a reduction in SPAD index, were consistent with crown gall disease^8,9,30^. Therefore, the differences observed between treatments in papaya plant growth are more likely attributable to the *Agrobacterium* treatment rather than to increased whitefly densities. Also, the impact of *A. tumefaciens* on plant physiology, including chlorophyll levels, could vary by *Agrobacterium* strain and host species^30^. Therefore, the present results may be specific to the strain used here, and different outcomes may be observed with other strains.

However, the precise cause of the more pronounced negative effects on plant parameters in the second trial compared with the first remains uncertain. Although this discrepancy may reflect experimental variability, other factors could be involved. Both trials shared the same inoculation date (July 12), but whitefly release began earlier in the first trial (August 14) than in the second (August 25), introducing an 11-day gap. This timing difference may have affected plant-pathogen-insect interactions. Whitefly feeding is known to activate the salicylic acid (SA) signaling pathway in host plants, which in turn may attenuate subsequent *Agrobacterium* infection^31^. Although gall formation occurred approximately one week later in the second trial compared to the first, this does not necessarily indicate that the plants were affected by *A. tumefaciens* at a later time^32^. Therefore, delayed infestation of whiteflies in the second trial might have limited the induction of such plant defenses, resulting in more severe symptoms of crown gall disease compared to the first trial. Furthermore, the results for canopy width, a plant parameter for which the difference between treatments became negligible during the later stages of the second trial, may also support this. Nevertheless, despite the negative effects of *A. tumefaciens* infection on several plant growth parameters and chlorophyll content, populations of *T. variabilis* were generally higher on infected plants than on controls. This pattern suggests that the relationship between plant physiological condition and whitefly population growth may not be straightforward in this system. A more detailed understanding of plant physiological condition at the time of infestation and its interaction with whitefly feeding is required to interpret these findings.

In conclusion, inoculating papaya plants with *A. tumefaciens* increased the density of *T. variabilis*, possibly associated with changes in plant physiology that may promote whitefly population growth. Although the treatment exhibited some negative effects on plant growth, particularly in the second trial, its impact on canopy structure was relatively limited. While *A. tumefaciens* has long been recognized for its gene-transfer capacity, its ecological effects on non-transgenic plant-insect interactions have received little attention. The present study provides empirical evidence that bacterial infection itself can influence herbivore population dynamics, expanding the current framework of plant-microbe-insect interactions beyond viral or fungal pathosystems.

## Supplementary Information

Below is the link to the electronic supplementary material.

Supplementary Material 1

Supplementary Material 2

## Data Availability

The data that support the findings of this study are publicly available at https://doi.org/10.5281/zenodo.17201268.
